# Levels, trends and socio-demographic determinants of infant and under-five mortalities in and around slum areas of Dhaka city, Bangladesh

**DOI:** 10.1016/j.ssmph.2022.101033

**Published:** 2022-01-28

**Authors:** Abdur Razzaque, Razib Chowdhury, AHM Golam Mustafa, Farzana Begum, Sohana Shafique, Alexander Lawton, Mohammad Zahirul Islam

**Affiliations:** a International Centre for Diarrhoeal Disease Research, (icddr,b), Bangladesh; b Fielding School of Public Health, University of California Los Angeles, USA; c Embassy of Sweden, Dhaka, Bangladesh

**Keywords:** Infant, Under-five, Mortality, Slum, Dhaka, Bangladesh

## Abstract

Infant and child mortality are often used to monitor the progress of national population health programs. The data for this study was collected from selected urban slums where icddr,b has maintained the Health and Demographic Surveillance System (HDSS). Using the HDSS database, 6,666 married women were selected and interviewed in 2018 to collect data on socioeconomic status, pregnancy history and safe motherhood practices. The study examined levels and trends of infant and under-five mortality for three periods: 1990–1999 (Period 1), 2000–2009 (Period 2), and 2010–2018 (Period 3) and examined socio-demographic differentials of infant and under-five mortality for Period 3. From Period 1 to Period 3, under-five mortality declined by 68.2%, with child mortality (1–4 years) declining more than infant mortality (84% vs. 65%). In the regression models for Period 3, infant and under-five mortality were higher for working than non-working mothers (infant: OR = 1.35*, CI: 0.98, 1.86; under-five: OR = 1.34*, CI: 0.99, 1.82), lower for girls than boys (infant: OR = 0.77*, CI: 0.57, 1.03; under-five: OR = 0.77*, CI: 0.58, 1.03), higher for small-size than normal/big-size babies (infant: OR = 4.11***, CI: 3.00, 5.64; under-five: OR = 3.68***, CI: 2.70, 5.02), higher for babies delivered vaginally than by caesarean section (infant: OR = 1.79**, CI: 1.14, 2.97; under-five: OR = 1.87***, CI: 1.21, 2.88), higher for babies delivered with complications than no complication (infant: OR = 2.16***, CI: 1.48, 3.15; under-five: OR = 2.21***, CI: 1.55, 3.18), and higher for babies born after a short (<24 months) birth interval (infant: OR = 1.71*, CI: 0.96, 3.05; under-five: OR = 1.63*, CI: 0.93, 2.86) than firstborns. While substantial progress has been made in reducing under-five and infant mortality, neonatal mortality have declined less slowly. Targeted population health interventions addressing the socio-demographic drivers of infant mortality, with a focus on the urban poor, will help Bangladesh achieve Sustainable Development Goal 3.

## Highlights

Infant and under-five mortalities were higher for:

• Working mothers than non-working
• Small-size babies than normal-size
• Vaginally delivered babies than caesarean section
• Babies delivered with complication than without
• Higher for babies born after a short birth interval than firstborns.

## 1 Introduction

Infant and child mortality are often used as indicators to monitor national population health programs and to examine weaknesses within health systems (McGuire, 2006). People in less developed countries tend to have less access to health services than those in developed countries. Irrespective of national development, the poor have less access to health services, resulting in mortality differentials (Peters et al., 2008). In recent years, differences in mortality by socioeconomic status have received renewed attention globally (SDG, 2019; Sridhar, 2019). To better understand how to best address this disparity, more studies are needed in diverse settings across multiple countries.

Although substantial progress has been made in achieving Millennium Development Goal 3 (reducing infant and child mortality), 5.4 million children under five died in 2017; of these deaths, 2.5 million were in the neonatal period, 1.6 million were in the post-neonatal period, and 1.3 million occurred between ages one and four. If the current mortality rate remains unchanged, 56 million children under five will die by 2030 (UNIGME, 2018). To address this issue, Sustainable Development Goal 3 states that countries should aim to reduce neonatal mortality to at least 12 per 1,000 livebirths and under-five mortality to at least 25 per 1,000 livebirths by 2030 (SDG, 2019).

In Bangladesh, under-five mortality has declined appreciably in recent decades, from 143.8 per 1,000 livebirths in 1990 to 32.4 in 2017 (UNICEF, 2018); however, the neonatal mortality rate is among the highest in the world (UNICEF Bangladesh, 2018). Moreover, under-five mortality among slum dwellers is about 50% higher than the national average (Razzaque, Chowdhury, & Mustafa, 2018). Bangladesh faces major challenges in achieving SDG 3 and national goals of improving maternal and child health, both of which have been made more difficult by a rapid increase in the slum population.

Numerous studies have documented the effects of parental socioeconomic status on infant and child mortality. (Caldwell, 1979; Gubhaju, Streatfield, & Majumder, 1991; Hobcraft, Mc Donald, & Rustein, 1984; Kim, 1988). Maternal education has emerged as one of the strongest predictors of child mortality, though other factors like women's autonomy, income, parental working status, standard of living index, household size, place of residence, and water/sanitation have also been shown to have an effect (Gokhale, Rao, & Garole, 2002; Hobcraft et al., 1984; Sastry, 1996). Caldwell (1984) argued that maternal education plays an important role in determining child survival, even after controlling for socioeconomic factors, a claim that has been further supported by other studies (Cleland & Van Ginneken, 1988; Boerma & Bicego, 1992; Cleland & Streatfield, 1992). Birth interval also plays a significant role on infant and child survival (DaVanzo, Hale, Razzaque, & Rahman, 2008; Gubhaju, 1986; Pebley & Millman, 1986); the probability of dying is much higher when the interval between births is short. Maternal age at birth also plays an important role in child mortality; the optimal childbearing age is between 20 and 34 years (Bhandari, Mandowara, Agarwal, & Jagdev, 1988; Hobcraft, McDonald, & Rutstein, 1985). In rural areas of south Asia, northern states of India, Pakistan, and Bangladesh, mortality rates of female children are often much higher than in male children (D'Souza & Chen, 1980; Dyson & Moor, 1982). Studies have also shown childhood mortality to be highest in a mother's first pregnancy and lowest in her 2nd and 3rd pregnancies (Dyson & Moor, 1982; Miller, 1993). Maternal and childhood immunization and health care utilization, including antenatal, postnatal, and delivery practices, also affect infant and child survival (Breiman et al., 2004; Ezeh, Agho, Dibley, Hall, & Page, 2015). Maternal health care is most effective if the visits are started in the early stages of pregnancy and continue at regular intervals throughout the pregnancy and postnatal period (Baqui et al., 2013).

Sustainability improves quality of life, protects the ecosystem, and preserves natural resources; this has become a major concern for policymakers across the world (Murshed et al., 2021; Rehman et al., 2021; Zakaria, Khan, Tan, Alvarado, & Dagar, 2022). Jairaj, Deka, Martin, and Kumar (2017) reported that implementation of renewable energy is likely to improve infant and child survival in poor communities by providing employment opportunities, ultimately leading to poverty reduction; this pattern is also expected to be true for the slum population.

Although a large number of studies have examined socio-demographic differentials of infant and under-five mortality in Bangladesh (Abir, Agho, Page, Hilton, & Dibley, 2015; Chowdhury, Hanifi, Mia, & Bhuiya, 2017; Chowdhury, Islam, & Hossain, 2010), none of these studies have specifically examined mortality differentials among slum dwellers. The rapidly increasing slum population of Bangladesh and stark differences in the health service infrastructure of urban versus rural areas (Kamal, Curtis, Hasan, & Jamil, 2016) warrants examination of the socio-demographic drivers of infant and under-five mortality in slums. The data for this study came from selected slums where icddr,b has maintained the Health and Demographic Surveillance System (HDSS), which was used for sample selection and background data. The objective of the study was to examine levels, trends, and socio-demographic differentials of infant and under-five mortality of selected slums in and around Dhaka city.

## 2 Methodology

### 2.1 Study area

The data for this study came from selected slums in and around Dhaka city, where, since 2015, icddr,b has maintained the HDSS for a population of 125,000. People living in urban slums face significant health risks due to limited access to public health infrastructure and overcrowding, which can promote disease transmission (Kamal et al., 2016). In these slums, 82% of households possess one bedroom, 95% households use pipe water for drinking and washing, 30% have a sanitary latrine with a sewage or septic tank, and slightly over 50% use a gas stove for cooking. Sharing of water sources (92%), latrines (90%), and kitchen (60%) is common and use of electricity for light is universal. Most households have an electric fan (96%) and a mobile phone (85%); 60% of households have television or *khat* (Razzaque, Chowdhury, & Mustafa, 2019).

In rural areas of Bangladesh, health services are often more structured than in urban areas. To improve the health status of the urban poor, the Local Government Division has implemented the Urban Primary Health Care Project which contracts the delivery of primary health care services to urban local government bodies and NGOs (UPHCSDP, 2019). Moreover, public and private hospitals and drug stores are often close to slum areas compared to rural settings. Despite the availability of health services (public, private, and NGOs), the use of maternity care services is low. It was reported that in 2017, 20% of pregnant women did not attend any antenatal check-ups, only 38% had four or more antenatal check-ups (Razzaque et al., 2018), and 55% percent did not have any postnatal visits.

### 2.2 Data sources

For routine HDSS data collection, fifteen female field workers conduct household visits every three months to collect information on birth, death, migration, marriage/divorce, and maternity care. Because the female field workers visit each household every three months, they maintain a good relationship with the community and with the household members. These female field workers were also trained to collect data for this study. To collect data on pregnancy history, we used the Bangladesh Demographic and Health Survey questionnaire, which is highly tested and widely used. Because the study area was located in the HDSS area, respondents were familiar with the survey questionnaire. Using the HDSS sampling frame, 6,800 married women (15–49 years) were randomly selected, of whom 6,666 were successfully interviewed in 2018 to collect data on socioeconomic status, pregnancy histories and safe motherhood practices.

Women were asked to provide a history of their live births, including sex of the child, date of birth, age of the child at the time of survey, survival status, and age at death (if applicable). Safe motherhood and delivery practice data were collected for births occurring in the most recent period (Period 3), including the number of antenatal visits, delivery type (vaginal or caesarean section), presence of complications during delivery (any complication), and reported size of the newborn (small and normal/big, determined by showing a picture). Birth interval categories (<24, 24–35 and 36+ months) were determined based on earlier studies (Cleland & Sathar, 1984; Koenig, Phillips, Campbell, & D'Souza, 1990; Rutstein, 2003). Individual and household socioeconomic data (e.g. possession of household assets, women's working status, education) were also collected/updated during the survey.

A total of 11,456 singleton births that occurred from 1990 to 2018 were analyzed; 820 of them died in the first five years of life. Infant and under-five mortality were initially examined for three periods: 1990–1999 (Period 1), 2000–2009 (Period 2), and 2010–2018 (Period 3). A detailed analysis of the socio-demographic differentials of mortality was conducted for births occurring in Period 3.

### 2.3 Statistical analyses

The dependent variables used in this study are: a) infant mortality – the probability of dying within the first year of life (died or survived); and b) under-five mortality – the probability of dying after birth and before one's fifth birthday (died or survived). Both bivariate (percent/rate) and multivariate (logistic regression) analyses were performed. All independent variables included in the model have been shown to have an effect on infant and under-five mortality in the literature. The following variables were included in the model: the mother's age at birth, sex of the child, mother's education status, household assets, mother's working status, number of antenatal visits, baby's size at birth, delivery type (vaginal or caesarean), presence of complications during delivery, and birth interval. Birth order was excluded from the model because it was highly collinear with birth interval and was not significant in bivariate analysis. For this study, results with p < 0.10 were considered statistically significant. SPSS version 20 was used for all analyses.

## 3 Results

**Fig. 1:**
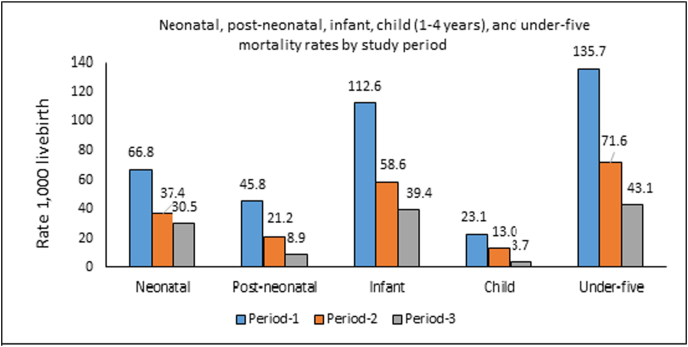
Neonatal, post-neonatal, infant, child (1-4 years), and under-five mortality rates by study period.

Fig. 1 shows neonatal, post-neonatal, infant, child, and under-five mortality rates for the three study periods. As expected, all mortality rates (per 1,000 live births) were higher in Period 1 (infant: 112.6; under-five: 135.7) than in Period 2 (infant: 58.6; under-five: 71.6) or Period 3 (infant: 39.4; under-five: 43.1).

**Fig. 2:**
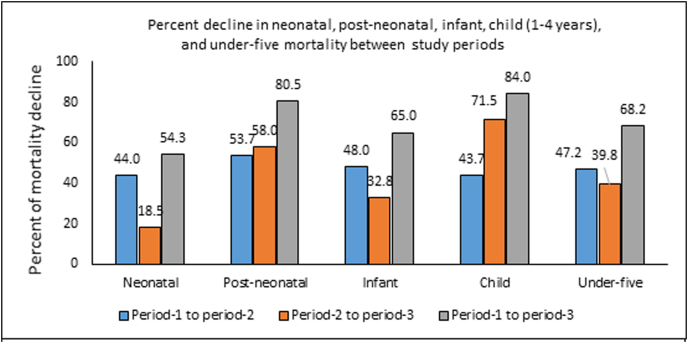
Percent decline in neonatal, post-neonatal, infant, child (1-4 years), and under-five mortality between study periods.

Fig. 2 shows the percent decline of each mortality type between study periods. All types of mortality declined over the study period, although the magnitude of decline varied (Fig. 2). From Period 1 to Period 2, neonatal mortality declined by 44%, post-neonatal declined mortality by 53.7%, infant mortality declined by 48%, child mortality (1–4 years) declined by 43.7%, and under-five mortality declined by 47.2%. Declines in all types of mortalities were also observed from Period 2 to Period 3, however, the rate of decline was much lower for neonatal (18.5%), infant (32.8%), and under-five (39.8%) mortality but higher for post-neonatal (58.0%) and child (71.5%) mortality compared to the changes observed from Period 1 to Period 2. Under-five mortality declined substantially (68.2%) over the whole study period (Period 1 to Period 3), while child mortality declined more than infant mortality (84% vs 65%).

**Fig. 3:**
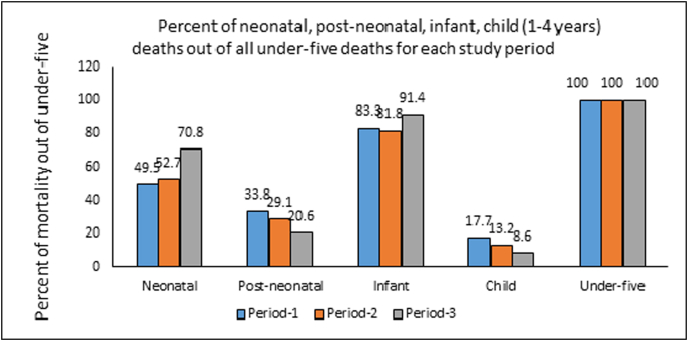
Percent of neonatal, post-neonatal, infant, child (1-4 years) deaths out of all under-five deaths for each study period.

Fig. 3 shows the percent of neonatal, post-neonatal, infant, and child deaths out of all under-five deaths for the three periods. Over the entire study period, the percent of neonatal deaths increased, while the percent of post-neonatal and child deaths decreased. In Period 1, 49.5% of all under-five deaths occurred in the neonatal period, which increased to 52.7% in Period 2 and 70.8% in Period 3. Over time, child mortality contributed less to under-five mortality, declining from 17.7% in Period 1, to 13.2% in Period 2, to 8.6% in Period 3. Thus, from Period 1 to Period 3, greater reductions in child mortality and post-neonatal mortality relative to neonatal mortality led to a high proportion of deaths occurring in neonatal period.

Table 1 shows differences in infant and under-five mortality by selected socio-demographic variables in Period 3. For both infant and under-five mortality, significant differences were found by mother's working status, birth interval length, survival status of previous child, size of baby at birth, number of antenatal visits, and presence of complication(s) during delivery.

**Table 1:** Infant and under-five mortality rate (per 1,000 births) by selected socio-demographic variables for births occurring in Period 3^+^.

Variables	Infant mortality (# of births)	P-value	Under-five mortality (# of births)	P-value
Age of mother at birth (years)				
<18	45.0 (489)		46.9 (490)	
18-24	41.2 (2499)		44.3 (2507)	
25 or more	36.3 (1845)	0.588	40.0 (1852)	0.705
Sex of child				
Boy	43.8 (2490)		46.8 (2498)	
Girl	35.4 (2343)	0.137	38.7 (2351)	0.163
Education of mother (years of schooling)				
0	41.1 (1339)		46.1 (1346)	
1-4	41.1 (1070)		42.9 (1072)	
5 or more	38.4 (2422)	0.889	41.1 (2431)	0.774
Mother's working status				
Not working	36.1 (3542)		39.1 (3553)	
Working	49.6 (1291)	0.034	53.2 (1296)	0.032
Household assets				
Low	42.1 (2163)		46.0 (2172)	
Medium	38.7 (904)		39.8 (905)	
Well-off	37.4 (1766)	0.744	40.6 (1772)	0.619
Birth interval				
First birth	43.0 (2213)		46.0 (2219)	
<24 months	73.0 (246)		73.0 (246)	
24–35 months	41.0 (315)		47.0 (317)	
36 or more months	32.0 (2059)	0.012	36.0 (2067)	0.036
Death of previous child				
Yes	55.6 (432)		62.1 (435)	
No	38.2 (4401)	0.078	41.0 (4414)	0.039
Size of baby at birth				
Normal/big	29.7 (4246)		33.1 (4261)	
Small	112.4 (587)	0.001	113.9 (588)	0.001
No. of antenatal visits				
0	51.8(1023)		58.2(1030)	
1-3	37.5(1867)		40.6(1872)	
4 or more	35.5(1943)	0.080	38.0(1947)	0.027
Type of delivery				
Vaginal	40.9 (3758)		44.3 (3771)	
Caesarean section	35.3 (1075)	0.405	38.0 (1078)	0.372
Complication(s) during delivery				
Yes	53.7 (1081)		58.0 (1086)	
No	35.7 (3752)	0.008	38.5 (3763)	0.005
All	39.7 (4833)		42.9 (4849)	

Note: ^+^Period 3 = 2010–2018; Chi-square test for levels of significance.

Table 2 shows multiple logistic regression estimates of selected variables for infant and under-five mortality for Period 3. Infant and under-five mortality were higher for working than non-working mothers (infant: OR = 1.35*, CI: 0.98, 1.86; under-five: OR = 1.34*, CI: 0.99, 1.82), lower for girls than boys (infant: OR = 0.77*, CI: 0.57, 1.03; under-five: OR = 0.77*, CI: 0.58, 1.03), higher for small-size than normal/big-size babies (infant: OR = 4.11***, CI: 3.00, 5.64; under-five: OR = 3.68***, CI: 2.70, 5.02), higher for babies delivered vaginally than by caesarean section (infant: OR = 1.79**, CI: 1.14, 2.97; under-five: OR = 1.87***, CI: 1.21, 2.88), higher for babies delivered with complications than without complications (infant: OR = 2.16***, CI: 1.48, 3.15; under-five: OR = 2.21***, CI: 1.55, 3.18), and higher for babies born after a short (<24 months) birth interval than firstborns (infant: OR = 1.71*, CI: 0.96, 3.05; under-five: OR = 1.63*, CI: 0.93, 2.86).

**Table 2:** Multiple logistic regression estimates of selected covariates for infant and under-five mortality for births occurring in Period 3^+^.

Covariates	Infant mortality Odds ratios (95% CI)	Under-five mortality Odds ratios (95% CI)
Mother's age at birth (years)		
18–24 (rc=<18)	0.97(0.60, 1.60)	1.02(0.63, 1.65)
25 or more (rc=<18)	0.88(0.49, 1.58)	0.91(0.51, 1.60)
Sex of child		
Girl (rc = Boy)	0.77*(0.57, 1.03)	0.77*(0.58, 1.03)
Mother's education (years of schooling)		
1–4 (rc = None)	1.02(0.67, 1.56)	0.94(0.63, 1.41)
5 or more (rc = None)	1.02(0.70, 1.47)	0.97(0.68, 1.38)
Mother's working status		
Working (rc = Not working)	1.35*(0.98, 1.86)	1.34*(0.99, 1.82)
Household assets		
Medium (rc = Low)	0.94(0.63, 1.43)	0.89(0.59, 1.32)
Well-off (rc = Low)	1.04(0.73, 1.49)	1.06(0.75, 1.49)
Birth interval (months)		
<24(rc = First birth)	1.71*(0.96, 3.05)	1.63*(0.93, 2.86)
24–35(rc = First birth)	0.88(0.46, 1.67)	0.93(0.51, 1.69)
35+(rc = First birth)	0.75(0.50, 1.13)	0.77(0.52, 1.13)
Previous child died	1.57*(0.97, 2.57)	1.60*(1.01, 2.55)
Yes (rc = No)		
Size of baby at delivery		
Small size (rc = Normal/big)	4.11***(3.00, 5.64)	3.68***(2.70, 5.02)
No. of antenatal visit		
1–3 (rc = 0)	0.74(0.50, 1.08)	0.71*(0.49, 1.02)
4 or more (rc = 0)	0.74(0.49, 1.12)	0.70*(0.47, 1.04)
Type of delivery		
Vaginal (rc = Caesarean section)	1.79**(1.14, 2.97)	1.87***(1.21, 2.88)
Complication(s) during delivery		
Yes (rc = No)	2.16***(1.48, 3.15)	2.21***(1.55, 3.18)
−2 Log Likelihood (df)	1509.21(17)	1626.13(17)

Note: ^+^Period 3 = 2010–2018; rc = Reference category; *p < 0.10, **p < 0.05, ***p < 0.01.

## 4 Discussion

In line with the global and national trends, our study found that infant and under-five mortality declined substantially over the last three decades among slum dwellers in Bangladesh. Over the study period (1990–2018), neonatal mortality declined to a much lesser extent (54.3%) than post-neonatal mortality (80.5%) and child (1–4 years) mortality (84%). Multivariate analysis for Period 3 births shows that infant and under-five mortality varied by the mother's working status, sex of the child, size of the baby, delivery type, presence of complication(s) during delivery, and birth interval. In bivariate analysis, significant differences were also seen based on the survival status of the previous born child and the number of antenatal visits, although these differences were no longer significant when controlling for the other factors included in the model.

Although under-five mortality declined appreciably over the study period, child mortality declined more than neonatal mortality, which is in consistent with national survey data (NIPORT 2020). Regarding the lower decline of neonatal versus child mortality in urban slums versus the rural Matlab HDSS area, use of antenatal care lower in slum areas than in the Matlab HDSS (government service area) (12.0% vs. 6.3%) and a higher proportion of deliveries occurred at home, attended by unskilled birth attendants (24.5% vs. 20.0%) (Haq et al., 2019; Razzaque et al., 2018). These trends are notable given the proximity of many types of health facilities (public, private, and NGOs) within the urban slums.

Although earlier studies reported that infant and under-five mortality varied by mother's age (Alam, 2000; Bhandari et al., 1988) and mother's education (Chowdhury et al., 2017; Uddin, Hossain, & Ullah, 2009) our study did not find such relationships, and in fact saw slightly higher mortality for boys than girls, the opposite of what had been observed previously (D'Souza & Chen, 1980). Using the same dataset for Period 1, Razzaque, Chowdhury, and Mustafa (2021a) reported that infant and under-five mortality differed by mother's age, mother's education, household assets, and sex of the child, while such differentials were not present in Period 3, including the opposite pattern for sex of the child. This change in the differentials of mortality is likely due to improvement of the country's socioeconomic conditions, which, along with the decline in the desired/actual number of children, has allowed couples to put more importance on the quality of their children's livelihood than on having more children. This has resulted in increased survival of children across all socio-demographic groups, with the biggest differences seen among the poorest group and among girls.

Infant and under-five mortality were higher for working than non-working mothers. Mothers who work outside the home cannot provide adequate care to the newborn (Titaley et al., 2008), which may increase the risk of neonatal death (Reid, 2001). Higher infant and under-five mortality among working mothers may also be due to working mothers having to perform other household duties in the family, limiting their ability to provide adequate child care (Ahmad, 2002).

In agreement with previous studies (Alam, 2000; DaVanzo et al., 2008; Gubhaju, 1986), we found that births that occurred after a short birth interval (<24 months) had higher infant and under-five mortality than firstborns or those born after a long birth interval. Alam (2000) reported that first-born children were at higher odds of dying in infancy than second births; physical immaturity may be of major importance in determining the relationship between teenage fertility and high neonatal mortality. Regarding birth interval, it has been reported that a short birth interval may lead to increased mortality through: i) growth retardation, low birth weight and endogenous factors; ii) reduction of mother's breast milk, and iii) low maternal care for the children (Plloni & Millman, 1985).

In this study, we found that the newborns of mothers who experienced complications during delivery had significantly higher mortality rates than those who did not experience complications. A previous study (Titaley et al., 2008) reported that newborns of mothers experiencing complications such as vaginal bleeding, fever, or convulsions during delivery, had significantly higher risks of dying than newborns delivered without complications. It has also been reported that newborns of mothers without severe delivery complications have better survival rates than newborns of mothers with eclampsia, intrapartum hemorrhage or longer labor duration (Mercer et al., 2006).

Our study found that infant and under-five mortality were lower for babies born by caesarean delivery than by vaginal delivery. Although caesarean delivery is considered to be a common procedure that can mitigate risks to the mother and neonate (Ghahiri & Khosravi, 2015; Malloy, 2008), concerns around overuse have been raised. In Bangladesh, the rate of caesarean section is over 50% higher than the WHO-recommended limit. Ye et al. (2015) reported that rates of caesarean section higher than 10% are not associated with decreased maternal or neonatal mortality, and thus may not be necessary. Our study found lower mortality for newborns delivered via caesarean section, which could be due to care received at the hospital after delivery, since mothers generally stay at the hospital for more time after a caesarian delivery (Razzaque et al. 2021c). It has been recommended that care should be provided to both the mother and baby by a skilled birth attendant during and immediately after birth, whether the birth takes place at facility or home (WHO/UNICEF, 2009). These services are particularly essential in the three days after birth, when mortality is exceptionally high (Baqui et al., 2013; Razzaque, Chowdhury, & Mustafa, 2021b).

Our study documented higher infant and under-five mortality for small than normal/big-size babies. Previous studies have reported significantly higher rates of neonatal mortality for preterm births compared to full term births (Baqui et al., 2013; Razzaque, Chowdhury, & Mustafa, 2021b), and higher rates for babies who were small for gestational age than those born in-term or normal for gestational age (Marchant et al., 2012; Ray, Park, & Fell, 2017). In a hospital-based study in Nepal, Ashish, Joha, Nelin, Clark, and Målqvist (2015) reported high neonatal mortality for babies that were both preterm and small for gestational age, followed by babies who were born preterm and normal size for gestational age.

Our study found that, from Period 1 to Period 3, post-neonatal and child mortality declined more than neonatal mortality; however, the causes of death were quite different within each period. Using data from the Bangladesh Demographic and Health Survey, Arifeen et al. (2005) reported that serious infections (31%) and acute respiratory problems (21%) were responsible for most neonatal deaths, while among children (1–4 years), serious infection (37%), injuries (22%), acute respiratory infection (17%), malnutrition (11%) and diarrhea (9%) were the top causes of mortality. We did not collect causes of death in this study.

## 5 Strengths and limitations of the study

Data for this study came from selected slums, where HDSS has been in operation since 2015. HDSS field workers are highly trained and motivated and visit each household every three months for routine data collection. HDSS field workers also collected the data for this survey based on HDSS sampling frame. Despite high quality data collection, the study did have some limitations.

As many of the women interviewed had a long pregnancy history, it's possible that recall lapse occurred; however, the field workers were adequately trained to minimize such errors. Furthermore, for households those migrated from a rural area, some children may not have been born or died in the slum. However, because the analysis of the differentials of infant and under-five mortality used data only from the most recent period, such cases are likely minimal.

Some variables were not infant-specific, such as the mother's working status and education; however, their changes over time may not be significant. The study excluded multiple births and births in which the mother died. This may underestimate the true mortality level; however, the frequency of these cases is likely small.

A future study using HDSS birth registration data that follows infants for survival and records cause of death data would help overcome these limitations. Such a study would allow for a more complete assessment of the socio-demographic differentials of infant and child mortality for slum dwellers.

## 6 Policy implications

Although infant and under-five mortality declined appreciably over the study period, mortality rates remain high in comparison to global averages, with a disparity between slum and non-slum dwellers. Infant deaths comprised 92% of all under-five deaths in Period 3, with deaths occurring in neonatal period for 70% of all under-five deaths. To accelerate reductions in infant and under-five mortality, targeted health interventions that focus on promoting the health of the mother in the prenatal period and of the newborn within the neonatal period are recommended. Promoting use of institutional medical services may hold particular value.

## 7 Conclusions

Although under-five mortality has declined appreciably over time, neonatal mortality remains very high and slum dwellers suffer from higher mortality rates than non-slum dwellers. Mortality rates varied considerably by mother's working status, sex of the child, size of the baby, delivery type, delivery complication, and birth interval. Targeted public health interventions program addressing these issues will help to achieve Sustainable Development Goal 3 for slum dwellers and for Bangladesh as a whole.

## Author's statement

AR designed the study. AR, RC, and AHMGM prepared the dataset and were involved in the preliminary analysis. AR and RC prepared the first draft of the manuscript. All authors participated in editing the manuscript and approved the final version for submission. The corresponding author had full access to all data and had the final responsibility for the decision to submit for publication.

## Declaration of competing interest

The authors declare that they have no competing interests.
